# Longitudinal study of primary progressive aphasia in a patient with pathologically diagnosed Alzheimer’s disease: a case report

**DOI:** 10.1186/s13256-021-02867-6

**Published:** 2021-05-26

**Authors:** Masahiko Takaya, Kazunari Ishii, Kazumasa Saigoh, Osamu Shirakawa

**Affiliations:** 1 Department of Neuropsychiatry, Faculty of Medicine, Kindai University, 377-2, Onohigashi, Osakasayama, Osaka, 589-8511 Japan; 2 Department of Radiology, Faculty of Medicine, Kindai University, Osaka, Japan; 3 Department of Neurology, Faculty of Medicine and Department of Clinical Genetics, Faculty of Medicine, Kindai University, Osaka, Japan

**Keywords:** Alzheimer’s disease, Nonfluent/agrammatic variant, Primary progressive aphasia, ^11^C-Pittsburgh compound-B, ^18^F-THK5351, Positron emission tomography, Standard language test of aphasia

## Abstract

**Background:**

Alzheimer’s disease is a neurodegenerative disease involving the deposition of pathologic amyloid-β and tau protein in the cerebral cortex. Alzheimer’s disease is commonly characterized by progressive impairment of recent memory. Primary progressive aphasia is also often observed in patients with Alzheimer’s disease. Moreover, language-associated symptoms, such as primary progressive aphasia, are diverse and varied in Alzheimer’s disease. However, nonfluent/agrammatic variant primary progressive aphasia is not generally considered a symptom of Alzheimer’s disease. To date, there has been no longitudinal study of primary progressive aphasia in Japanese-speaking patients or in patients speaking other languages with pathologically diagnosed Alzheimer’s disease. Here we present a longitudinal study of primary progressive aphasia in a Japanese patient pathologically diagnosed with Alzheimer’s disease.

**Case presentation:**

A 75-year-old Japanese man, whose wife reported that his memory was impaired, also suffered from suspected aphasia. He was pathologically diagnosed with Alzheimer’s disease using ^11^C-Pittsburgh compound-B positron emission tomography and ^18^F-THK5351 positron emission tomography. Based on clinical observation and the results of the Japanese standard language test of aphasia, he was also diagnosed with nonfluent/agrammatic variant primary progressive aphasia. During the subsequent 2 years, his cognitive impairment, aphasia, and behavioral and psychological symptoms of dementia progressed. Furthermore, progression of pathologic amyloid-β and tau protein deposition was revealed through ^11^C-Pittsburgh compound-B positron emission tomography and ^18^F-THK5351 positron emission tomography. Although the results of [^123^I] iodoamphetamine single-photon emission computed tomography suggested corticobasal degeneration, this was not observed on the [^123^I] FP-CIT single-photon emission computed tomography (SPECT) (DaTscan). A previous study had reported that Alzheimer’s disease with a nonfluent/agrammatic variant primary progressive aphasia was accompanied by corticobasal degeneration; however, this was not true in our case.

**Conclusions:**

This is possibly the first longitudinal study of nonfluent/agrammatic variant primary progressive aphasia in a Japanese-speaking patient with pathologically diagnosed Alzheimer’s disease, but without corticobasal degeneration.

## Background

The amyloid cascade hypothesis postulates that Alzheimer’s disease (AD) is a neurodegenerative disease which is caused by the deposition of pathologic amyloid-β and tau protein in the cerebral cortex. Currently, the pathological criterion for AD is limited to significant accumulation of pathologic amyloid-β deposits.

AD is commonly characterized by progressive recent memory impairment, alongside additional progressive symptoms such as visuospatial cognitive impairment, executive functional impairment, primary progressive aphasia (PPA), and behavioral psychiatric symptoms. Language-associated symptoms including PPA in AD are diverse and varied.

PPA can be classified into the following three types: nonfluent/agrammatic variant PPA (nfvPPA), semantic variant PPA (svPPA), and logopenic variant PPA (lvPPA) [1]. However, other types of PPA that cannot be classified into these three types (or a mixture of types) also exist [2].

Different variants of aphasia may reportedly appear in AD, as follows: 100% of lvPPA is due to AD, 25% of mixed PPA is due to AD, and 4% of nfvPPA is due to AD complicated by corticobasal degeneration (CBD) [2]. However, to the best of our knowledge, no longitudinal study of PPA in patients with pathologically diagnosed AD has been conducted to date. We present here a longitudinal study of nfvPPA in a Japanese-speaking patient pathologically diagnosed with AD.

## Case presentation

A 75-year-old Japanese man with suspected PPA visited our clinic, accompanied by his wife. He was a university graduate. He had a history of atrial fibrillation, which was controlled by a pacemaker, and diabetes, which was under control. His family medical history was not particularly remarkable. His family had recently observed memory impairment and stuttering in the patient, which prompted his visit to our clinic. During the medical interview, his speech was effortful with stuttering; however, inconsistent speech sound errors were not obvious.

The following tests were performed four times in total, at baseline, half a year after base line, 1 year after baseline, and 2 years after baseline (Table 1): Mini-Mental State Examination (MMSE), cognitive subscale of the Alzheimer’s Disease Assessment Scale Japanese version (ADAS-cog), frontal assessment battery, digit span of the Wechsler Adult Intelligence Scale–Third Edition (WAIS-III), logical memory subtest I of the Wechsler Memory Scale–Revised (WMS-R), logical memory subtest II of the WMS-R, copy of the Rey-Osterrieth complex figure test (ROCFT), delayed recall of the ROCFT, and the Japanese reading test. The Neuropsychiatric Inventory (NPI) was performed twice in interviews with his wife, both at 6 months and 2 years after baseline. The Japanese Raven’s Coloured Progressive Matrices (RCPM) was performed 6 months after baseline. The results are shown in Table 1. The results of the logical memory subtest and ROCFT indicated that the patient’s verbal memory was severely impaired at baseline, and his visual memory impairment at baseline showed gradual worsening, respectively. The results of the MMSE and ADAS-cog showed worsening of general cognitive impairment. His behavioral and psychological symptoms of dementia also showed progression, as revealed by the results of the NPI. The RCPM was performed once 6 months after the baseline; the patient scored 26 out of 36, which indicated nonverbal cognitive impairment [3].

**Table 1:** The results of neurocognitive tests

	Baseline	6 months after baseline	1 year after baseline	2 years after baseline
MMSE (out of 30)	14	12	11	6
ADAS-cog (out of 70)	29.7	36	39	52
FAB (out of 18)	9	8	7	5
WMS-R				
Logical memory I (out of 50)	0	3	0	0
Logical memory II (out of 50)	0	0	0	0
WAIS-III				
Digit span				
Forward (out of 16)	6	5	5	3
Backward (out of14)	5	4	2	2
ROCFT				
Copy (out of 36)	36	30	31	22
Delayed recall (out of 36)	7	6	8	0
JART (out of 50)	8	4	3	1
NPI (frequency × severity)	N/A	Apathy: 4 × 2 Abnormal eating: 4 × 2 Each of the other subtests: 0 × 0	N/A	Agitation/Aggression: 2 × 2 Depression: 2 × 1 Apathy: 4 × 3 Euphoria: 1 × 2 Abnormal eating: 1 × 2 Each of the other subtests: 0 × 0

*ADAS-cog* cognitive subscale of the Alzheimer‘s Disease Assessment Scale Japanese version, *FAB* frontal assessment battery, *JART* Japanese reading test, *MMSE* Mini-Mental State Examination, *NPI* Neuropsychiatric Inventory, *RCPM* Japanese Raven’s Coloured Progressive Matrices, *ROCFT* Rey-Osterrieth complex figure test, WAIS-III Wechsler Adult Intelligence Scale–Third Edition, *WMS-R* Wechsler Memory Scale–Revised

The Japanese Standard Language Test of Aphasia (SLTA) was also performed at each of the four time points [4, 5]. The SLTA was developed in Japan and consists of 26 items for evaluating aphasia; the test kit is commonly used and easily available in Japan [4, 5]. The results of the SLTA are shown in Table 2. Scores worsened over the 2 years for all of the following subtests: auditory comprehension (ability to obey verbal commands), naming, verbal explanation of behavior of persons and movement of others in pictures, verbal explanation of Manga, verbal fluency, reading comprehension of short sentences (ability to point at pictures), reading comprehension (ability to obey written commands), writing words with Kana (ability to represent pictures), writing explanation of Manga, dictation of Kana letters, dictation of Kanji words, dictation of Kana words, dictation of short sentences, and four arithmetic operations on paper. All other subtest scores were stable throughout the 2 years. Scores for auditory comprehension of words (ability to point at pictures), word repetition, reading aloud Kana letters, reading aloud Kana words, reading comprehension of Kanji words (ability to point at pictures), and reading comprehension of Kana words (ability to point at pictures) showed no errors during the 2 years.

**Table 2:** The results of all subtests of SLTA

Subtest	Baseline	6 months after baseline	1 year after baseline	2 years after baseline
(1) Auditory comprehension of words (to point at pictures) (out of 10)	10	10	10	10
(2) Auditory comprehension of short sentences (to point at pictures) (out of 10)	9	10	7	7
(3) Auditory comprehension (to obey verbal commands) (out of 10)	10	7	4	0
(4) Auditory comprehension (to point at Kana letters) (out of 10)	9	10	10	10
(5) Naming (out of 20)	14	12	10	4
(6) Word repetition (out of 10)	10	10	10	10
(7) Verbal explanation of behavior of persons and movement of others in pictures (out of 10)	10	9	6	5
(8) Verbal explanation of Manga (one to six stages)	4	4	5	1
(9) Sentence repetition (out of 5)	3	3	2	2
(10) Verbal fluency (number of words)	8	3	2	2
(11) Reading aloud Kanji words (out of 5)	5	5	5	3
(12) Reading aloud Kana letters (out of 10)	10	10	10	10
(13) Reading aloud Kana words (out of 5)	5	5	5	5
(14) Reading aloud short sentences (out of 5)	5	5	5	3
(15) Reading comprehension of Kanji words (to point at pictures) (out of 10)	10	10	10	10
(16) Reading comprehension of Kana words (to point at pictures) (out of 10)	10	10	10	10
(17) Reading comprehension of short sentences (to point at pictures) (out of 10)	10	9	10	6
(18) Reading comprehension (to obey written commands) (out of 10)	10	5	7	0
(19) Writing words with Kanji (to represent pictures) (out of 5)	1	0	0	0
(20) Writing words with Kana (to represent pictures) (out of 5)	4	3	1	0
(21) Writing explanation of Manga (one to six stages)	3	1	1	N/A
(22) Dictation of Kana letters (out of 10)	10	6	6	0
(23) Dictation of Kanji words (out of 5)	3	2	1	1
(24) Dictation of Kana words (out of 5)	3	1	0	N/A
(25) Dictation of short sentences (out of 5)	4	1	0	N/A
(26) Four arithmetic operations on paper (out of 20)	11	6	6	2

*SLTA* Japanese Standard Language Test of Aphasia, *N/A* the patient could not perform the task

Briefly, the results of the SLTA showed impaired comprehension of syntactically complex sentences, whereas single word comprehension and object knowledge were spared. Additionally, the content of his speech during the SLTA subtest of verbal explanation of Manga showed that his agrammatism in language production had progressed. These clinical symptoms met the criteria for a clinical diagnosis of nfvPPA [1].

**Fig. 1:**
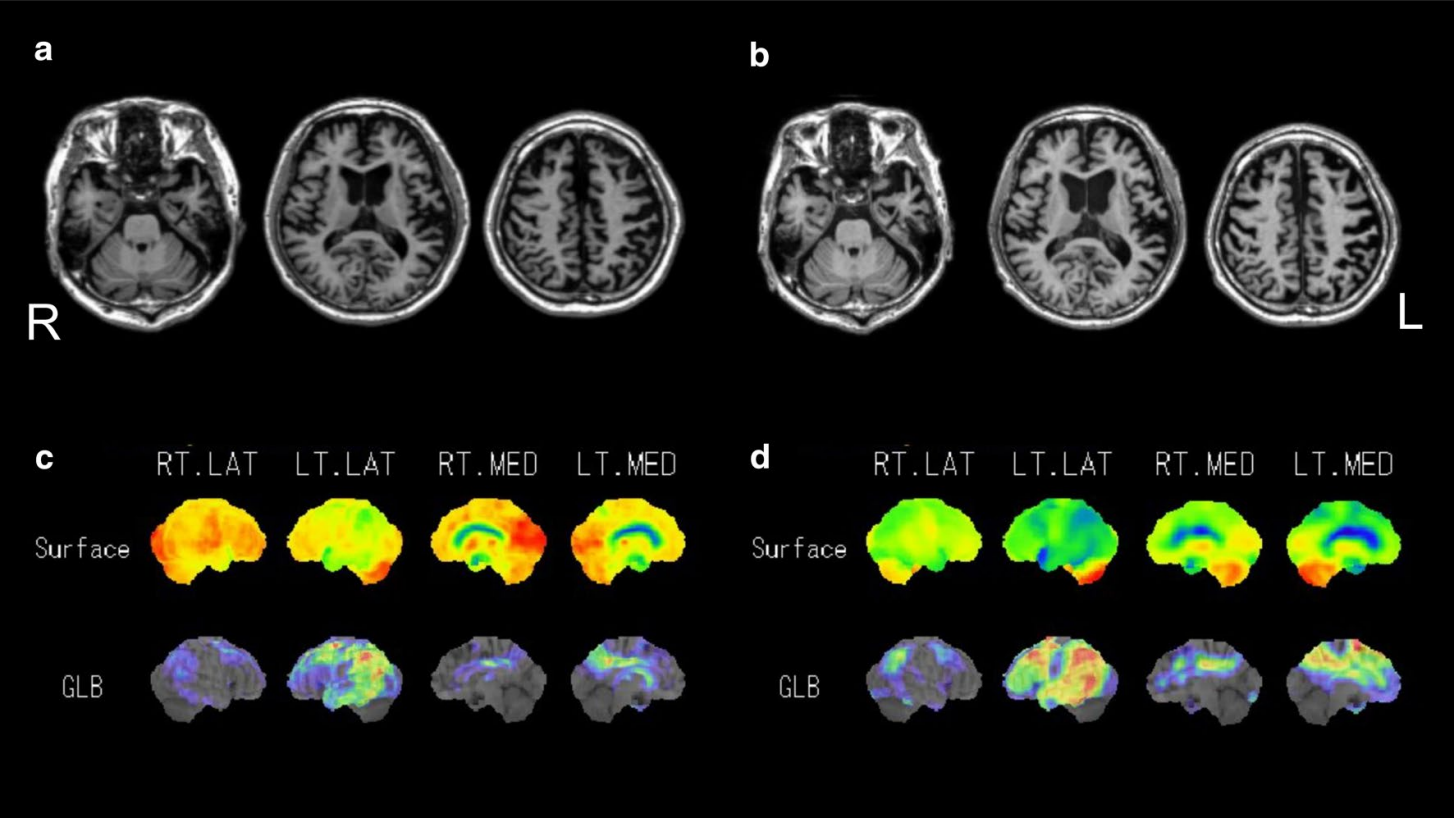
**a** and **b** are magnetic resonance images at baseline and 2 years after baseline, respectively. **c** and **d** are [^123^I] iodoamphetamine single-photon emission computed tomography images showing hypoperfusion at baseline and 2 years after baseline, respectively, using statistical voxel-based analysis using three-dimensional stereotactic surface software.

**Fig. 2:**
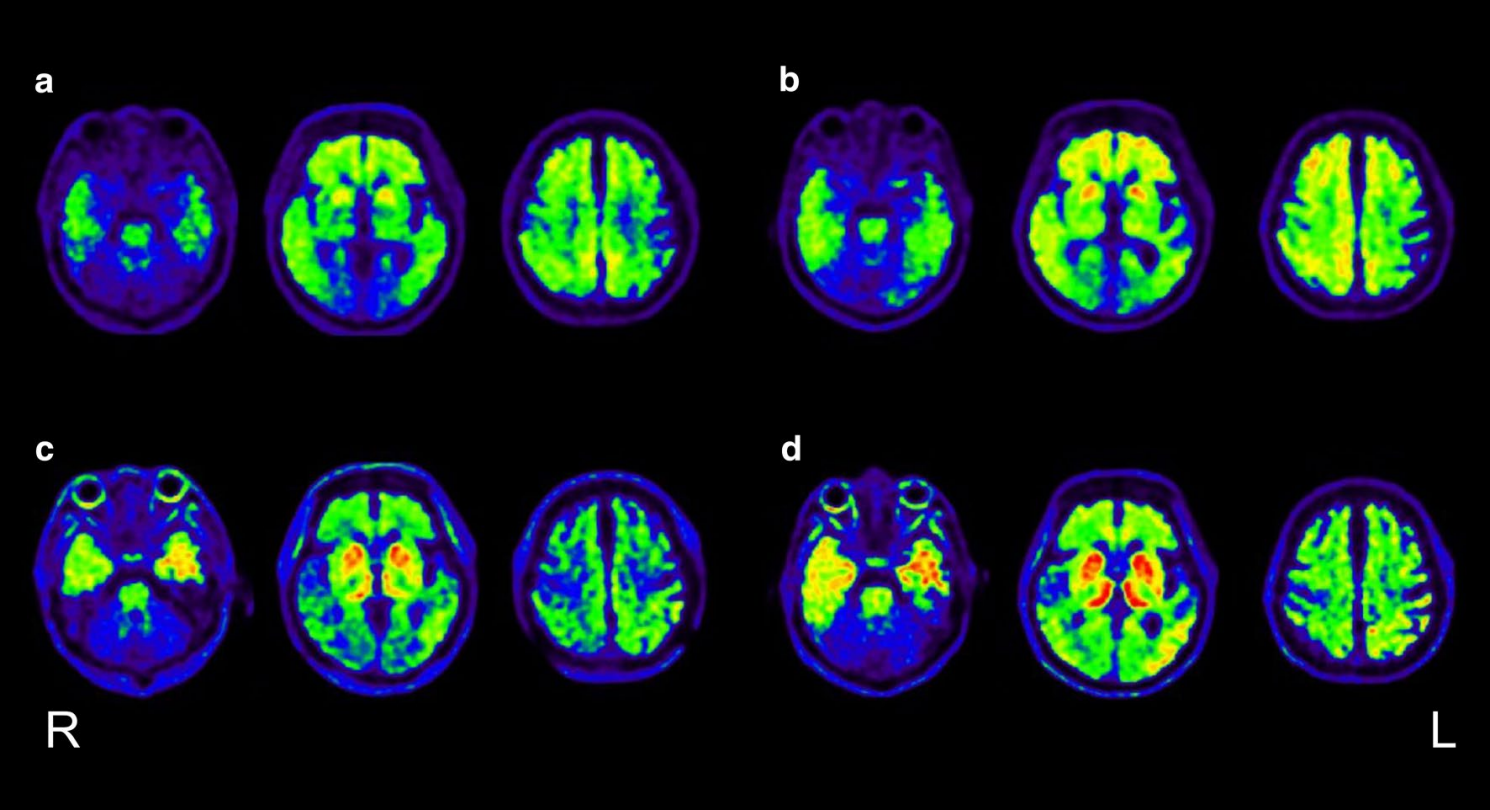
**a** and **b** are ^11^C-Pittsburgh compound-B positron emission tomography images at baseline and 2 years after baseline, respectively. **c** and **d** are ^18^F-THK5351 positron emission tomography images at baseline and 2 years after baseline, respectively.

The results of the magnetic resonance imaging (MRI), [^123^I] iodoamphetamine single-photon emission computed tomography (^123^I-IMP SPECT), ^11^C-Pittsburgh compound-B (PiB) positron emission tomography (PET), and ^18^F-THK5351 (THK5351) PET at baseline and 2 years are shown in Figs. 1 and 2. The MRI results showed global cerebral atrophy, which progressed during the 2 years (Fig. 1). PiB-PET and THK5351 PET, which indicated pathologic amyloid-β and tau protein deposition, did not show obvious laterality (Fig. 2); however, ^123^I-IMP SPECT imaging showed significant regional hypoperfusion dominantly in the left hemisphere, particularly in the bilateral parietal and temporal association cortex, the precuneus, the posterior cingulate cortex, the frontal association cortex, and the occipital lobe, which suggested CBD (Fig. 1). These figures showed that pathologic amyloid-β and tau protein deposition progressed during the 2 years, and that hypoperfusion also progressed during this period. The finding of ^18^F-2-fluoro-2-deoxy-d-glucose (FDG) PET at baseline was almost the same as that of ^123^I-IMP SPECT, that is, glucose uptake was decreased dominantly in the left hemisphere, particularly in the bilateral parietal and temporal association cortex, the precuneus, the posterior cingulate cortex, the frontal association cortex, and the occipital lobe, which also suggested CBD. There were no remarkable findings on either [123I] FP-CIT SPECT (DaTscans), which was performed twice.

Collectively, taking into account all results, the patient was diagnosed with nfvPPA with definite AD [1]. The patient also met the DSM5 (Diagnostic and Statistical Manual of Mental Disorders, Fifth Edition) criteria for AD [6]. The patient’s clinical features included occasional urinary incontinence, progression of personality and behavioral changes based on the NPI, limb-kinetic apraxia at around 6 months after baseline, some inability to recognize grandchildren and old friends at around 18 months after baseline, and progression of ideomotor apraxia. A score of 3 was found on the modified ranking scale both 6 months and 2 years after baseline. These clinical symptoms did not meet the criteria for CBD [7]. Neither the patient nor his spouse expected to receive any pharmacotherapy. We did not introduce speech and language therapy because it would have been painful for the patient. However, we advised the couple to use an elderly adult day-care center, and the patient regularly went to such a center near their home.

## Discussion

We present the first longitudinal case study of nfvPPA in a patient with pathologically diagnosed AD. We observed nfvPPA in a patient who was pathologically diagnosed with AD based on PiB PET and THK5351 PET evaluations. This is not commonly observed; a previous study by Spinelli *et al*. reported that there were cases of nfvPPA with AD and CBD but not with AD alone [2]. Furthermore, nfvPPA has recently been considered an exclusion criterion for probable AD dementia [8]. This discrepancy may be because the criteria for agrammatism differ between Japanese and US/UK researchers owing to their distinct grammar systems.

To the best of our knowledge, this is the first longitudinal study of a Japanese-speaking patient with PPA observed in pathologically definite AD, using SLTA. Aphasia in AD is not well understood and is often only discussed with other neurocognitive impairments that appear in AD. Only a few studies have investigated aphasia in detail using SLTA in only clinically diagnosed AD [5]. Furthermore, PPA in neurodegenerative dementias other than AD have been investigated longitudinally using SLTA in only a few studies [9].

The results of both [^123^I] FP-CIT SPECTs (DaTscans) indicated that there was no CBD. However, there have been studies of dopamine transporter binding in autopsy-confirmed CBD, one of which reported that it is possible that patients with CBD have delayed neuronal loss in the substantia nigra [10]. Thus, our [^123^I] FP-CIT SPECT (DaTscan) results cannot be used to rule out CBD. However, considering that clinical features did not yet fully meet the criteria for CBD, our results also do not indicate CBD in our patient, although we believe that he might progress to CBD in the future. This is because the results of IMP SPECT, FDG PET, and nfvPPA suggested CBD. This type of progression is a feature of some dementias caused by neurodegenerative diseases. For example, patients who are diagnosed with AD initially sometimes progress to dementia with Lewy bodies.

Radiation to the skull from nuclear medicine imaging and cardiovascular risk factors, including atrial fibrillation, can precipitate the onset of dementia in the elderly. Also, diarrhea and subsequent frailty following internal exposure might lead to dementia, in which case dementia may be prevented by probiotic supplementation [11].

Our study had several limitations: (1) we presented only one case of aphasia progression in a Japanese-speaking patient with AD, and (2) we used the SLTA to evaluate aphasia, including agrammatism. The SLAT, which was developed during predecessor conferences of the Japan Society for Higher Brain Dysfunction, is only available in Japanese and is commonly used only in Japan [4]. Because Japanese grammar is very different from that of other languages, the definition of “agrammatism” is also very different. Therefore, though we hope that the SLTA will be standardized to enable its use to evaluate aphasia in non-Japanese-speaking people in both clinical and research settings, it would be too difficult to standardize the SLTA.

## Conclusion

We present the first longitudinal study of nfvPPA in a Japanese-speaking patient with pathologically diagnosed AD; we were not able to find evidence of CBD in the patient.

## Data Availability

Data sharing is not applicable to this article because no data sets were generated or analyzed during the study.

## Abbreviations

AD: Alzheimer’s disease
ADAS-cog: The cognitive subscale of Alzheimer’s Disease Assessment Scale-Japanese version
CBD: Corticobasal degeneration
DSM5: Diagnostic and Statistical Manual of Mental Disorders, Fifth Edition
^123^I-IMP SPECT: [^123^I] Iodoamphetamine single-photon emission computed tomography
FDG: ^18^F-2-fluoro-2-deoxy-d-glucose
lvPPA: Logopenic variant primary progressive aphasia
MMSE: Mini-Mental State Examination
MRI: Magnetic resonance imaging
nfvPPA: Nonfluent/agrammatic variant primary progressive aphasia
PiB: ^11^C-Pittsburgh compound-B
PET: Positron emission tomography
PPA: Primary progressive aphasia
RCPM: Japanese Raven’s Coloured Progressive Matrices
SLTA: Japanese Standard Language Test of Aphasia
svPPA: Semantic variant primary progressive aphasia
THK5351: ^18^F-THK5351
WAIS-III: Wechsler Adult Intelligence Scale–Third Edition
WMS-R: Wechsler Memory Scale–Revised
